# Loss of HMG-CoA Reductase in *C. elegans* Causes Defects in Protein Prenylation and Muscle Mitochondria

**DOI:** 10.1371/journal.pone.0100033

**Published:** 2014-06-11

**Authors:** Parmida Ranji, Manish Rauthan, Christophe Pitot, Marc Pilon

**Affiliations:** Department of Chemistry and Molecular Biology, University of Gothenburg, Gothenburg, Sweden; Oregon Health and Science University, United States of America

## Abstract

HMG-CoA reductase is the rate-limiting enzyme in the mevalonate pathway and the target of cholesterol-lowering statins. We characterized the *C. elegans hmgr-1(tm4368)* mutant, which lacks HMG-CoA reductase, and show that its phenotypes recapitulate that of statin treatment, though in a more severe form. Specifically, the *hmgr-1(tm4368)* mutant has defects in growth, reproduction and protein prenylation, is rescued by exogenous mevalonate, exhibits constitutive activation of the UPR^er^ and requires less mevalonate to be healthy when the UPR^mt^ is activated by a constitutively active form of ATFS-1. We also show that different amounts of mevalonate are required for different physiological processes, with reproduction requiring the highest levels. Finally, we provide evidence that the mevalonate pathway is required for the activation of the UPR^mt^.

## Introduction

The mevalonate pathway converts acetyl-CoA into small prenylated lipids that are in turn precursors for many essential metabolites [1], [2]. In mammals, the mevalonate pathway is essential for the biosynthesis of cholesterol, coenzyme Q (also known as ubiquinone; a component of the electron transport chain in mitochondria), dolichols (important for N-glycosylation of proteins) and isoprenoids (farnesyl pyrophosphate or geranylgeranyl pyrophosphate; critical for the membrane association of small GTPases). Because the mevalonate pathway is the source of cholesterol in mammals, it is the target of a class of compounds, the statins, that lower plasma cholesterol levels by inhibiting the rate-limiting enzyme in the pathway, namely 3-hydroxy-3-methyl-glutaryl-CoA (HMG-CoA) reductase, that converts HMG-CoA into mevalonate. Millions of patients use statins daily to lower their cholesterol levels, and hence reduce the risk of cardiovascular disease. The great majority of statin-treated patients experience no significant side effects, but some patients experience muscle pains or, in rare cases, muscle breakdown, and other side-effects have been reported [3]. Statins also have anti-inflammatory effects, and are promising anti-cancer drugs because they inhibit the prenylation of small GTPases, such as RAS, which are commonly activated in human cancers [4], [5]. Importantly, little is known about the mechanisms for many of the effects of statins that are unrelated to their cholesterol lowering action. One of our goals is therefore to understand specifically the effects of inhibiting the mevalonate pathway that are unrelated to the inhibition of cholesterol synthesis.

The mevalonate pathway is conserved in *C. elegans* except that worms lack the branch of the pathway that leads from farnesyl pyrophosphate to cholesterol [6]. Statins cause many phenotypes in *C. elegans*, including decreased protein prenylation, induction of the endoplasmic reticulum (UPR^er^), growth arrest, sterility and lethality depending on the developmental stage of the treated worms [7]. These effects are due to HMG-CoA reductase inhibition since they are abrogated by the addition of mevalonate to the culture medium. Activation of the mitochondrial unfolded protein response (UPR^mt^) leads to statin resistance in *C. elegans*, suggesting that mitochondrial insult is a critical consequence of statin treatment in this organism [8].

In the present study we sought to establish a genetic model to study the effects of mevalonate pathway inhibition in *C. elegans*. To this end, we characterized a *C. elegans* mutant, *hmgr-1(tm4368)*, that lacks the HMG-CoA reductase gene. This mutant recapitulates many of the phenotypes that are induced by statins, and allowed us to make several novel observations.

## Results

### Domain Structure of the *C. elegans* HMGR-1 Protein

**Figure 1:**
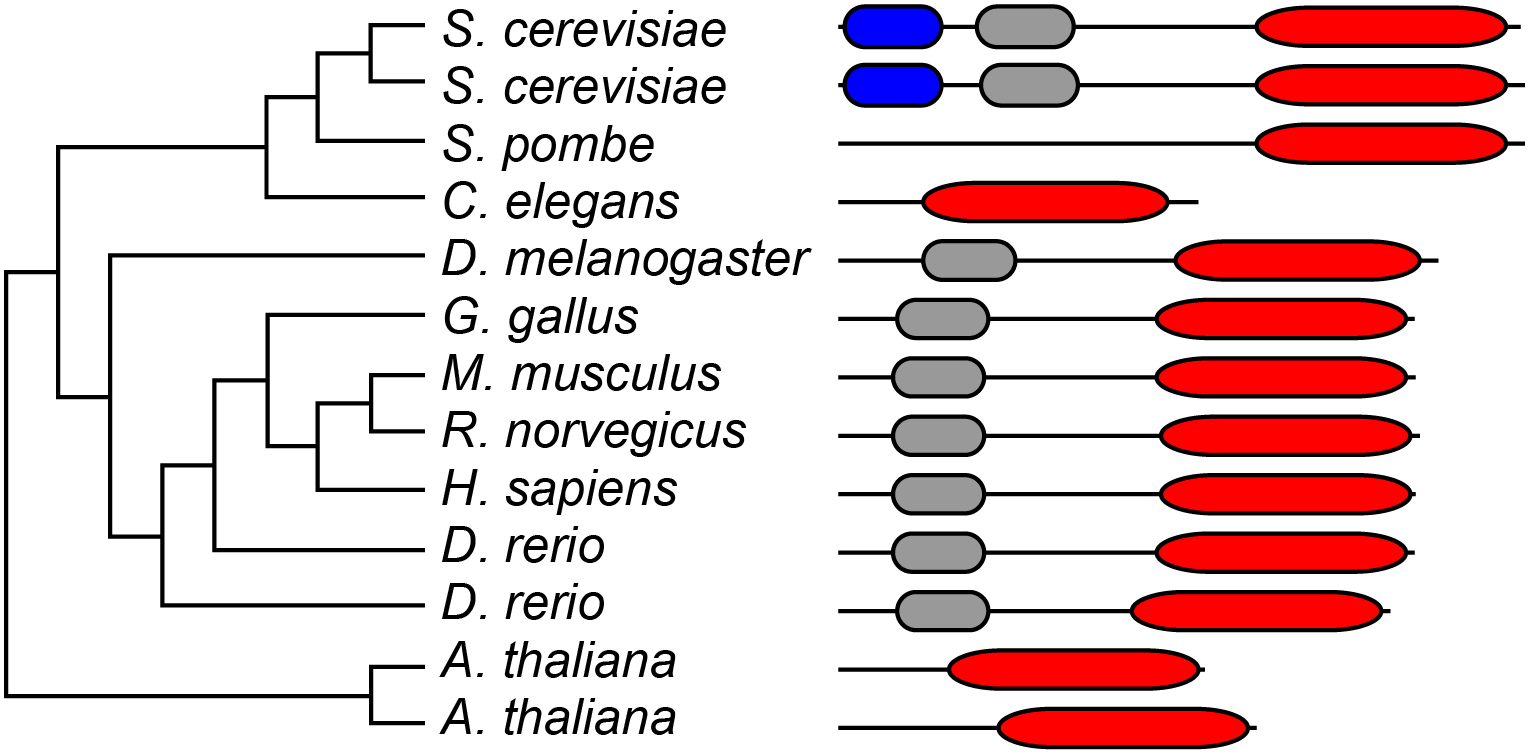
TreeFam cladogram of HMG-CoA reductases. The TreeFam family HMGCR (3-hydroxy-3-methylglutaryl-Coenzyme A reductase, TF105362) has 117 entries from 104 species. The final alignment was 1717 AA long and on average 56% conserved. TreeBest [9] was used to build a tree and reconcile it with the species tree. BLUE: N-terminal domain with HPIH motif found in fungi and with unknown function. GRAY: Sterol-sensing domain of SREBP cleavage-activation [26]. This domain is absent in *C. elegans*. RED: HMG-CoA reductase catalytic domain.

The *hmgr-1* gene encodes the sole homolog of HMG-CoA reductase in *C. elegans.* A sequence comparison using TreeFam shows that nematodes have lost the sterol-binding domain during the course of evolution: the worm HMGR-1 protein contains a 373 amino acid-long HMG-Co A reductase enzymatic domain but lacks the sterol-binding domain of SREBP cleavage-activation that is present in baker’s yeast, fruit fly and vertebrates (**Fig. 1**) [9] [10]. *C. elegans* HMGR-1 is therefore probably not regulated by sterol abundance, which is consistent with the fact that the mevalonate pathway does not contribute to cholesterol synthesis in this organism [6].

### The *Hmgr-1(tm4368)* Mutant can be Rescued with Mevalonate

**Figure 2:**
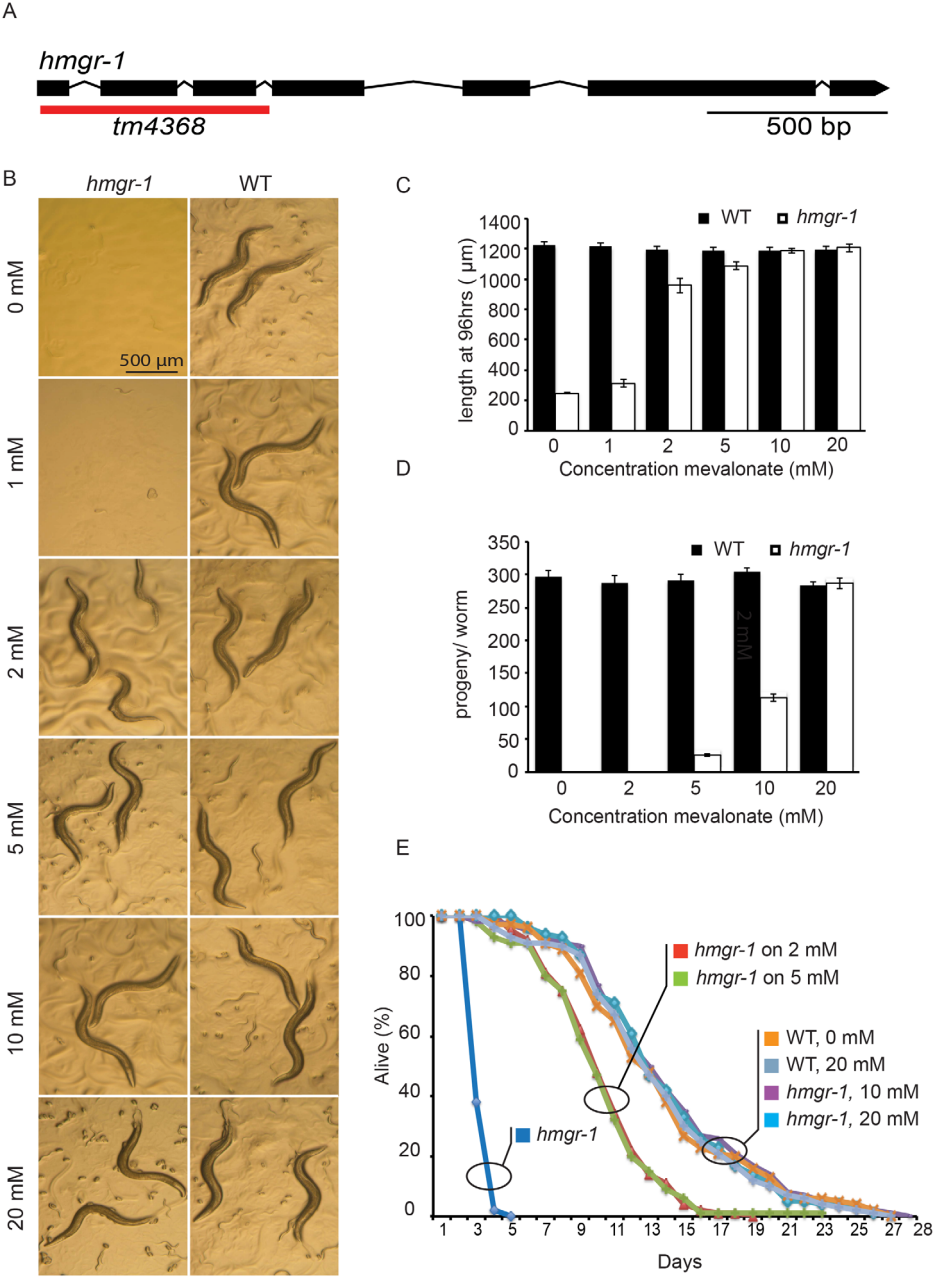
The *hmgr-1(tm4368)* mutant is rescued by exogenous mevalonate. (A) Structure of the *hmgr-1* gene; the region deleted in the *tm4368* allele is underlined in red. (B-E) Effect of various concentrations of mevalonate on wild-type and *hmgr-1(tm4368)* mutant worms showing representative images 96 hours after deposition of L1s onto new plates (B), length (C), total brood size (D) and life span (E).

The *hmgr-1(tm4368)* deletion mutant lacks all but the first seven nucleotides of the first exon and lacks both exons 2 and 3 in their entirety, with the deletion ending in the middle of the third intron (**Fig. 2A**). The deletion removes the first 25 amino acids within the essential HMG-CoA reductase enzymatic domain, and an eventual transcript made from this mutant allele would carry a shifted open reading frame of 48 amino acids. The *tm4368* allele is therefore very likely a null allele, a conclusion that is consistent with our experimental results. In particular, the *hmgr-1(tm4368)* mutation is lethal on normal growth plates, but can be fully rescued by including 20 mM mevalonate (**Fig. 2B–E**). Testing the effects of different amounts of mevalonate led to the observation that while 2 mM is sufficient to rescue growth of L1s into adults, 10 mM is required to restore a normal life span, and 20 mM is required to restore full reproductive potential. This suggests that several different physiological processes are dependent on the availability of mevalonate, with reproduction requiring the highest concentration.

### The *hmgr-1(tm4368)* Mutant Exhibits Constitutively Activated UPR^er^ but not UPR^mt^

**Figure 3:**
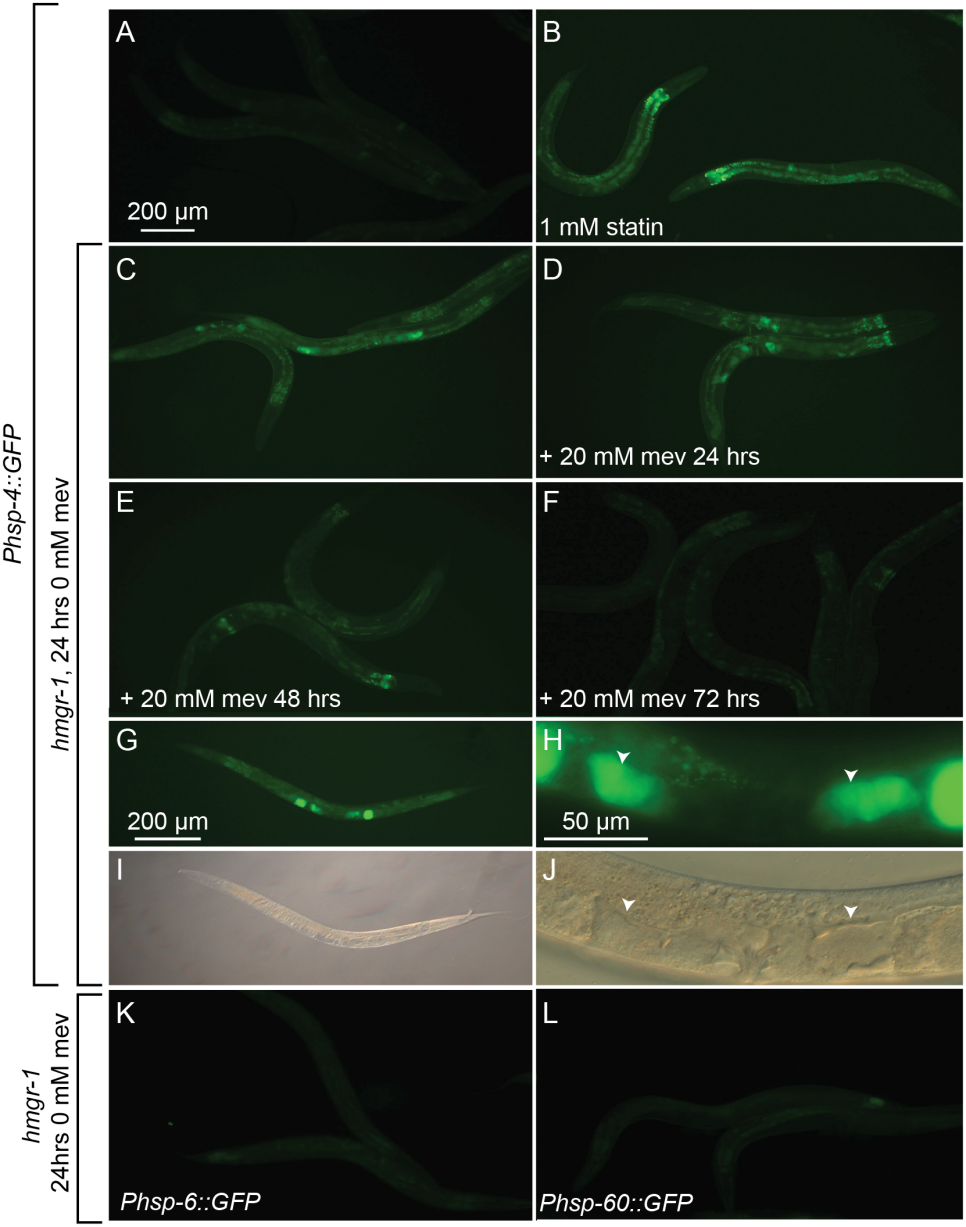
The UPR^er^ but not the UPR^mt^ is activated in the *hmgr-1(tm4368)* mutant. (A–B) The UPR^er^ reporter *Phsp-4::GFP* is expressed in wild-type L4 worms exposed to 1 mM fluvastatin for 24 hours. (C–D) *Phsp-4::GFP* is also expressed in *hmgr-1(tm4368)* mutant L4s deprived of mevalonate for 24 hours, but is silenced within 72 hours when these worms are subsequently provided 20 mM mevalonate. (G–J) *hmgr-1(4368)* mutants frequently expressed high levels of *Phsp-4::GFP* in what appear to be disorganized embryos on either side of the spermatheca (two are indicated by arrowheads in H). (K–L) The UPR^mt^ reporters *Phsp-6::*GFP and *Phsp-60::*GFP are not expressed in the *hmgr-1(tm4368)* deprived of mevalonate for 24 hours.

We have previously shown that inhibiting HMGR-1 in *C. elegans* using statins results in the activation of the endoplasmic reticulum unfolded protein response (UPR^er^) but not that of the UPR^mt^. The *hmgr-1(tm4368)* mutation recapitulates these pharmacological effects of statins: the UPR^er^ reporter *Phsp-4::GFP* is strongly activated in the mutant grown without mevalonate while two UPR^mt^ reporters, *Phsp-6::GFP* and *Phsp-60::GFP* are not (**Fig. 3**). Interestingly, *hmgr-1(tm4368)* worms grown without mevalonate for 24 hours will completely silence the UPR^er^ reporter when provided with mevalonate for 72 hours. This indicates that the ER stress caused by the absence of mevalonate is reversible. The *hmgr-1(tm4368)* mutants also occasionally show strong UPR^er^ in disorganized embryos on either side of the spermatheca (see **Fig. 3G–J**).

### The *hmgr-1(tm4368)* Mutant Exhibits Reversible Decreased Protein Prenylation

**Figure 4:**
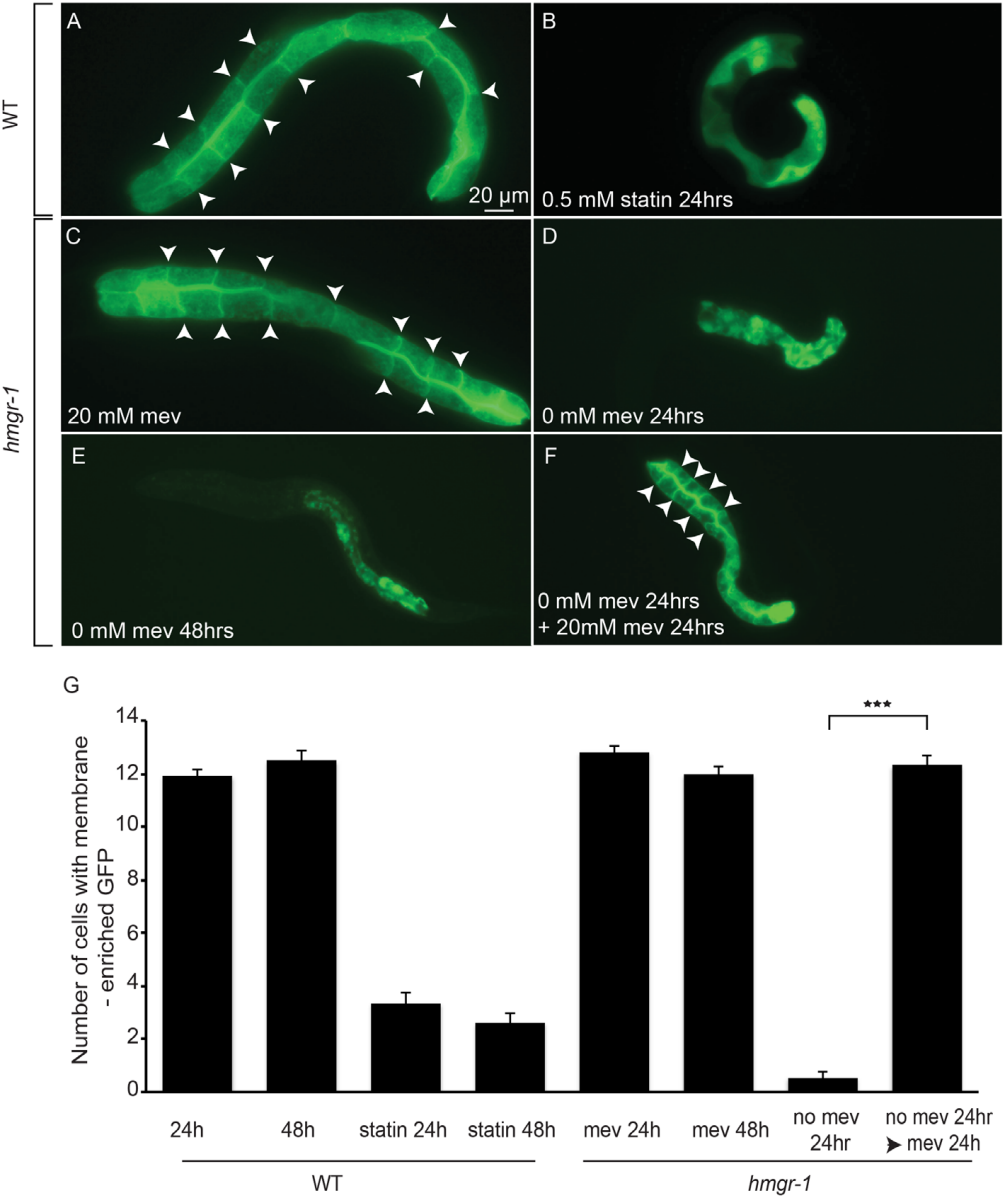
Exogenous mevalonate is essential for prenylation in the *hmgr-1(tm4368)* mutant. The prenylation reporter *pGLO-1P::GFP-CAAX* becomes diffusedly distributed in wild-type L1 worms exposed to 0.5 mM fluvastatins for 24 hours (A–B) or in *hmgr-1(4368)* mutants deprived of mevalonate for 24 or 48 hours (C–E). *hmgr-1(4368)* L1s deprived of mevalonate for 24 hours then provided with mevalonate for 24 hours show a clear restoration of prenylation (F). (G) Quantification of the degree of prenylation in the different genotypes and treatments. ***p<0.001 using Student’s *t-*test. Arrowheads indicate membrane enriched GFP.

Important outputs of the mevalonate pathway include prenylated lipids, namely farnesyl pyrophosphate (FPP) and geranylgeranyl pyrophosphate (GGPP), that are essential for the prenylation of small GTPases and other proteins. Prenylation causes the membrane association of these proteins, and is therefore required for their activity. We previously developed a prenylation reporter in which the CAAX C-terminal domain of *ras-2* is added to the GFP coding sequence such that the GFP becomes membrane-enriched when prenylated [7]. As with statin-treated worms, the *hmgr-1(tm4368)* mutant exhibits a prenylation defect that is evident from the diffused distribution of the prenylation reporter (**Fig. 4**). This phenotype is abrogated when the mutant is grown continuously in the presence of 20 mM mevalonate (**Fig. 4**), and is also reversible since mutants grown in the absence of mevalonate for 24 hours, and showing loss of prenylation, will become normalized within a further 24 hours of cultivation in the presence of mevalonate (**Fig. 4**).

### The *hmgr-1(tm4368)* Mutant Exhibits Irreversible Muscle Mitochondria Disorganization

**Figure 5:**
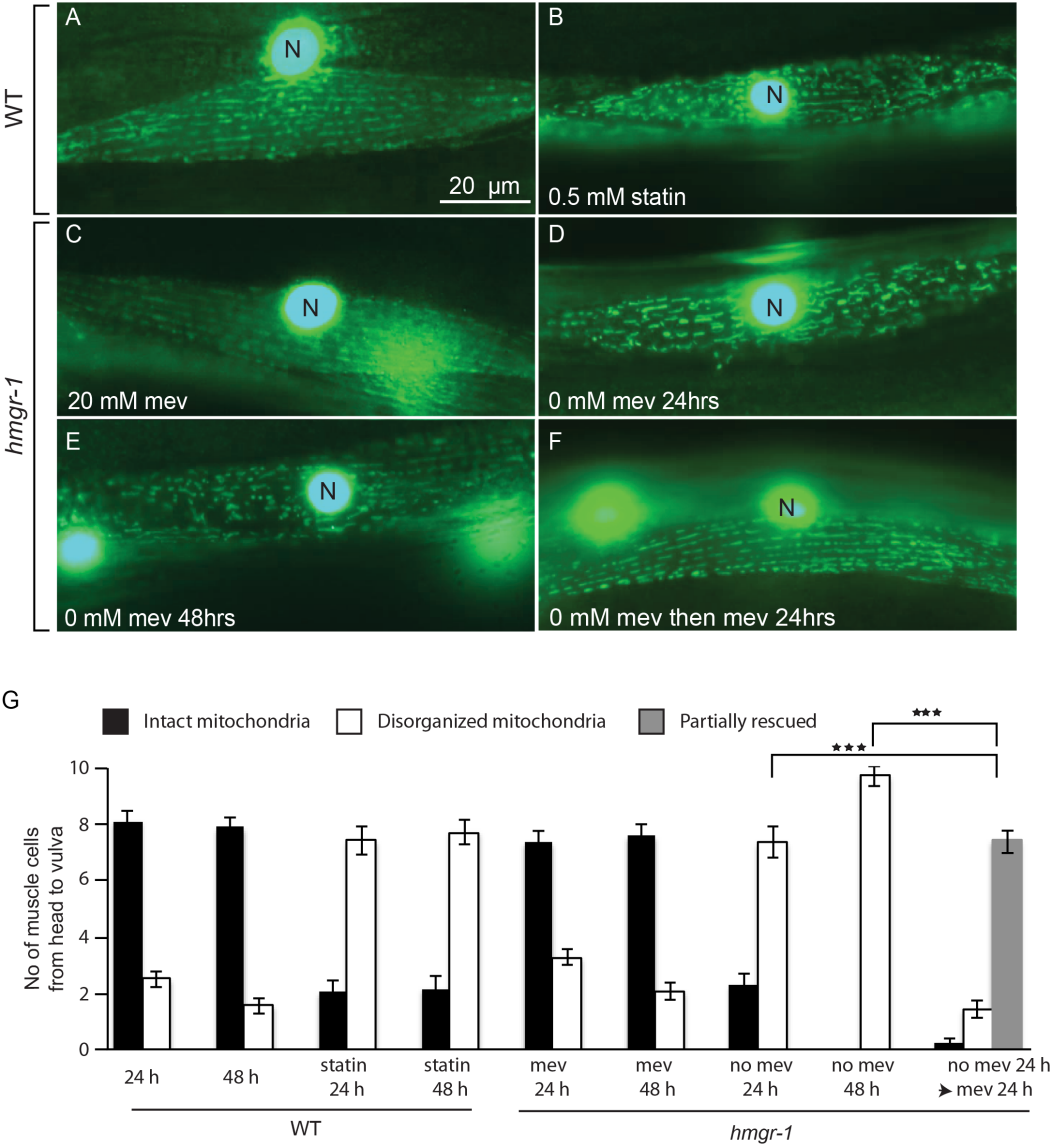
Exogenous mevalonate is essential for normal mitochondria morphology in the *hmgr-1(tm4368)* mutant. Wild-type worms show rows of evenly spaced mitochondria when grown on normal plates (A) but exhibit disordered mitochondria when grown on 0.5 mM fluvastatin (B). Similarly *hmgr-1(tm4368)* mutants grown on 20 mM mevalonate show rows of evenly spaced mitochondria (C) but exhibit disordered mitochondria when grown for 24 hrs or 48 hrs without mevalonate (D–E). *hmgr-1(tm4368)* mutants grown without mevalonate for 24 hours then provided 20 mM mevalonate for 24 hours show partially normalized mitochondria morphology (F). All worms in this figure were L4s at the start of the experiment and carry the transgene *ccIs4251* that contains GFP reporters showing the body muscle nuclei and the morphology of their mitochondria [27]. (G) Is a quantification of the mitochondria ordering where the number of muscle cells from head to vulva were counted and scored as having intact mitochondria (as in A), disorganized mitochondria (as in D) or partially rescued mitochondria (as in F). GFP-positive nuclei are labeled with “N”.

Patients receiving statin treatment occasionally experience muscle pains or, rarely, rhabdomyolysis [3], [11], and these effects can be reproduced in animal models, including in rats [12]. Using a GFP reporter expressed specifically in body wall muscle nuclei and mitochondria, we found that statin treatment also causes disorganization of the muscle mitochondria *C. elegans*, and that this effect could be prevented by the inclusion of 20 mM mevalonate in the culture medium (**Fig. 5A–C**). Similarly, the *hmgr-1(tm4368)* mutant exhibits obvious muscle defects when grown without mevalonate, and this is only partly reversible when mevalonate is supplied after a period of 24 hours of deprivation (**Fig. 5D–F**). *C. elegans* therefore also depends on a functional mevalonate pathway for maintenance of mitochondria organization in muscle.

### The *hmgr-1* Gene is Expressed Most Strongly and Consistently in Spermatheca and Pharyngeal and Vulva Muscles

**Figure 6:**
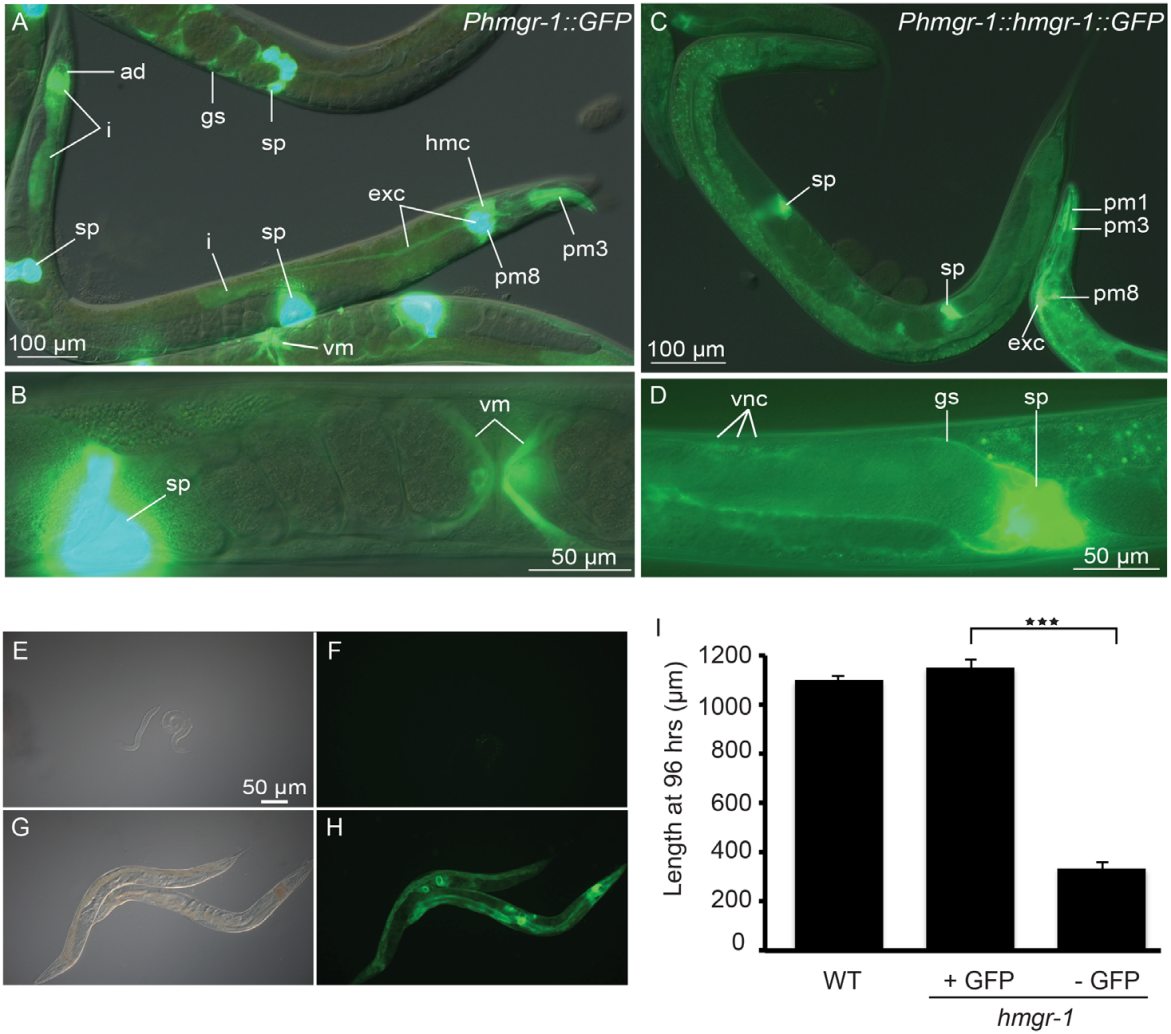
Several tissues express *hmgr-1* reporters. Expression of the Phmgr-1::GFP transcriptional reporter (A–B) and Phmgr-1::HMGR-1::GFP translational reporter (C–D). Structures labeled are as follows: ad (anal depressor), exc (excretory canal), gs (gonad sheath), hmc (head mesodermal cell), i (intestine), pm1, pm3 and pm8 (pharyngeal muscles 1, 3 and 8), sp (spermatheca), vm (vulva muscles), vnc (ventral nerve cord). (E–F) and (G–H) respectively show GFP-negative and GFP-positive progeny from *hmgr-1(4368); Ex[Phmgr-1::HMGR-1::GFP rol-6]* transgenic animals grown on normal plates, i.e. without exogenous mevalonate; the GFP-positive progeny grow as well as wild-type animals while the GFP-negative progeny do not grow (I). ***p<0.001 using Student’s *t-*test.

To examine the expression pattern of *hmgr-*1, we created a transcriptional reporter, *Phmgr-1::GFP*, and a translational reporter, *Phmgr-1::HMGR-1::GFP*. The transcriptional reporter is expressed in several tissues, but predominantly in spermatheca, excretory canal cell, vulva muscles, the pharyngeal muscles pm3 and pm8, the anal depressor and, more weakly, in the intestine (**Fig. 6A–B**). The translational reporter is strongest in the spermatheca, excretory canal cell and pharyngeal muscles, and is also expressed in the gonad sheath cell and the ventral nerve cord (**Fig. 6C–D**). These reporters likely provide an accurate view of *hmgr-1* expression given that the translational reporter effectively rescues the *hmgr-1(tm4368)* mutant (**Fig. 6E–I**).

### Activated ATFS-1 Rescues *hmgr-1* Mutants in Low Mevalonate Concentrations

**Figure 7:**
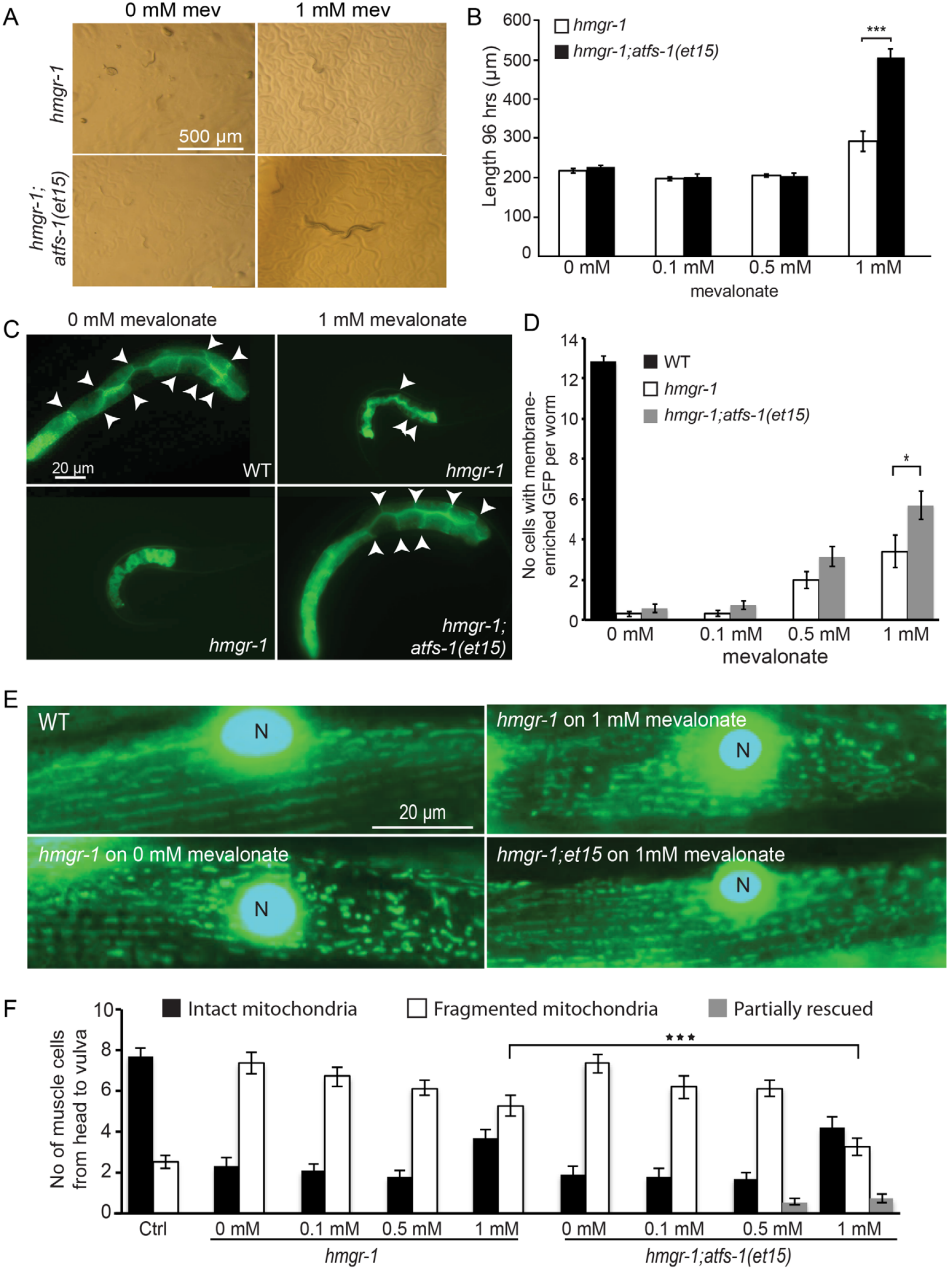
The *atfs-1(et15)* mutation suppresses *hmgr-1(tm4368)* mutant phenotypes when small amounts of mevalonate are provided. Length (A–B), membrane localization of the prenylation reporter *pGLO-1P::GFP-CAAX* (C–D) and mitochondria structure (E–F) are significantly improved by the addition of 1 mM mevalonate in *atfs-1(et15); hmgr-1(tm4368)* double mutants than in *hmgr-1(tm4368)* single mutants. GFP-positive nuclei are labeled with “N”. *p<0.05 and ***p<0.001 using Student’s *t-*test.

We previously showed that gain-of-function mutations in the UPR^mt^ activator ATFS-1 can protect worms against mevalonate pathway inhibition using statins [8]. ATFS-1 is a leucine zipper transcription factor that contains a mitochondrial targeting signal (MTS) at its N terminus and a nuclear localization signal at its C-terminus; during mitochondrial stress ATFS-1 is not efficiently targeted to mitochondria and instead accumulates in the nucleus to activate UPR^mt^ effectors [13]–[16]. Interestingly, the *atfs-1(gof)* mutants lacking a functional MTS did not show improved statin resistance when exogenous mevalonate was provided, which we interpreted as evidence that statins do not fully inhibit HMG-CoA reductase in those experiments [8]. Here, we used the *hmgr-1(tm4368)* allele to test this hypothesis: if *atfs-1(gof)* alleles acts by allowing *C. elegans* to survive and proliferate with residual output from the mevalonate pathway, then such alleles should improve the health and growth of *hmgr-1(tm4368)* null mutants provided with low doses of mevalonate. **Fig. 7** shows that this is the case: 1 mM mevalonate, which is too low a dose to rescue the *hmgr-1(tm4368)* mutants, greatly improved the growth of *hmgr-1(tm4368); atfs-1(et15)* double mutants (**Fig. 7A–B**). Similarly, the double mutant showed an improved benefit from 1 mM mevalonate in terms of protein prenylation (**Fig. 7C–D**) and, to a weaker degree, also in muscle mitochondria morphology (**Fig. 7E–F**).

### The Mevalonate Pathway is Required for UPR^mt^ Activation

**Figure 8:**
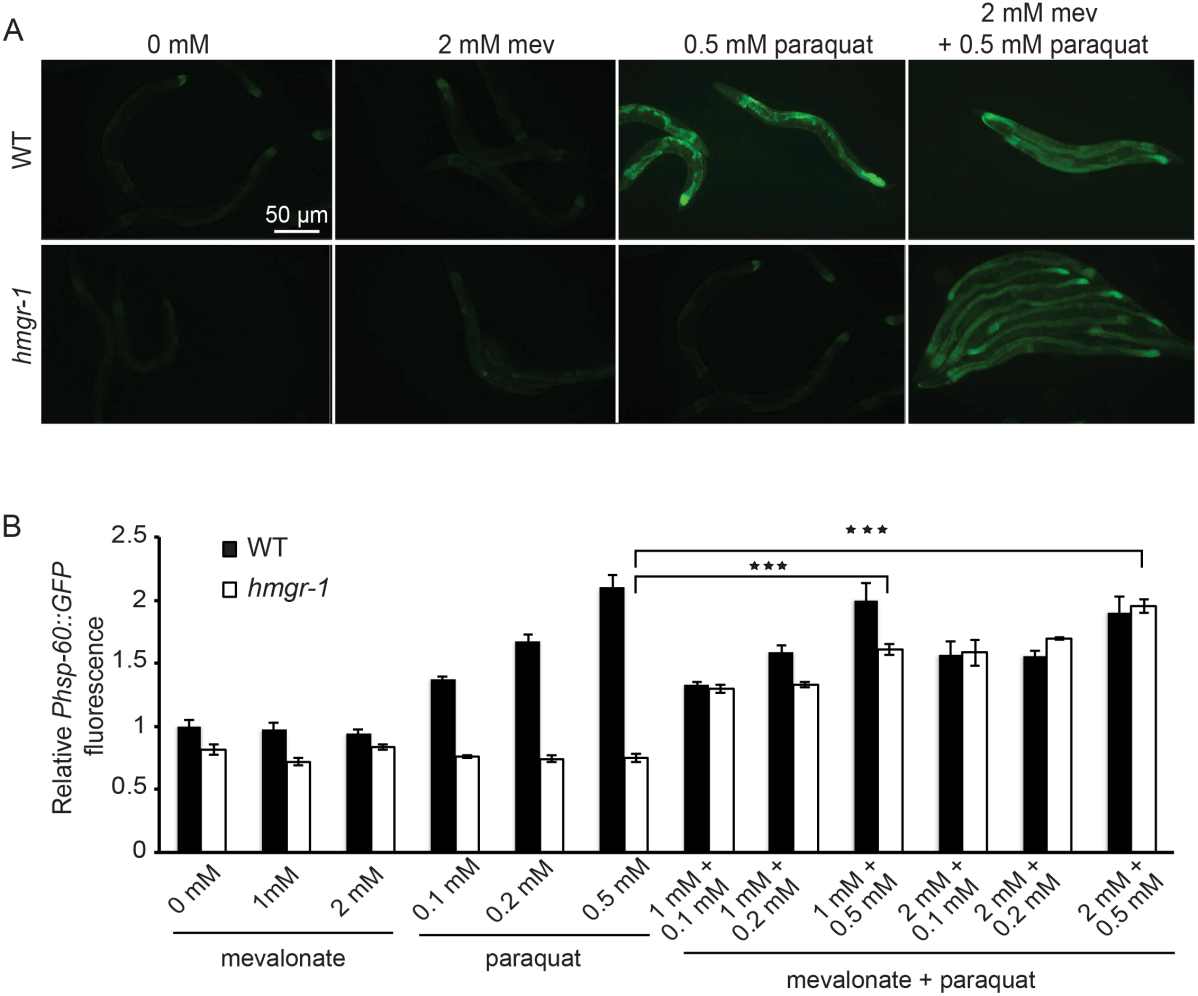
Mevalonate is required for UPR^mt^ induction. Synchronized L1s were grown for 24 hours on normal plates or, in the case of *hmgr-1(tm4368)*, plates containing 20 mM mevalonate, then transferred to experimental plates and scored after a further 48 hours. Note that paraquat does not cause UPR^mt^ activation in the *hmgr-1(tm4368)* mutant unless mevalonate is provided. ***p<0.001 using Student’s *t-*test.

We have shown that the UPR^mt^ response confers protection against a limited mevalonate supply. It is therefore intriguing that the *hmgr-1(tm4368)* mutant fails to activate this response. One possibility is that mevalonate is itself required for UPR^mt^ activation. This hypothesis was tested using paraquat, a known inducer of the UPR^mt^ in *C. elegans.* While wild-type worms show strong induction of the *Phsp-60::GFP* UPR^mt^ reporter when grown on 0.5 mM paraquat, the *hmgr-1(tm4368)* mutant shows no such induction unless small amounts of mevalonate are provided (**Fig. 8A–B**). This strongly suggests that mevalonate is required for UPR^mt^ activation. Note that small amounts of mevalonate supplied exogenously have no obvious effects on the *Phsp-60::GFP* expression in wild-type or in the *atfs-1(et15)* mutant (**Fig. S1**).

## Discussion

The *hmgr-1(tm4368)* recapitulates the effects of statins on *C. elegans* in several ways: it causes defects in growth, reproduction and protein prenylation, is rescued by exogenous mevalonate, exhibits constitutive activation of the UPR^er^ and require less mevalonate to be healthy when the UPR^mt^ is activated by a constitutively active form of ATFS-1. Additionally, we observed that worms lacking a functional *hmgr-1* develop severe defects in muscle mitochondria morphology and that these, as well as the protein prenylation defects, are reversible if mevalonate is provided within 24 hours.

We previously noted that the mitochondrial stress caused by statins was unusual since it did not result in the activation of the UPR^mt^, which would evidently be a suitable protective response as demonstrated by the fact that gain-of-function alleles of *atfs-1*, in which the UPR^mt^ is constitutive, are resistant to statins [8]. The explanation for this conundrum is now clear: our present work, and that of Liu at al. [17], show that inhibition of the mevalonate pathway prevents the activation of the protective UPR^mt^. It seems possible that one or more small GTPases, which depend on the mevalonate pathway for their membrane association via prenylation, may be required for UPR^mt^ activation. The hypothesis clearly has merit given that RNAi against the small GTPase RHEB-1 prevents UPR^mt^ activation in *C. elegans* [18], and that the mammalian homolog of RHEB-1, Rheb, is an important regulator of the mTOR Complex 1 (mTORC1) which has important roles in maintaining mitochondria homeostasis [19].

Our study of the *hmgr-1* mutant suggests that different concentrations of mevalonate may be required for different physiological processes: growth is completely rescued with as little as 2 mM, life span requires 10 mM and reproduction requires 20 mM. The mevalonate pathway is responsible for the production of many important metabolites, and the enzymes involved in the different branches of the pathway have Kms that vary greatly [6], [20]. Thus, different concentrations of mevalonate will differentially rescue the various mevalonate pathway branches, with production of FPP used for farnesylation of small GTPases being the easiest to rescue. Given the important roles of small GTPases in many essential cellular processes ranging from cytoskeletal regulation to organelle homeostasis, it is likely that growth is critically dependent on their function, which is also the easiest to rescue with low amounts of mevalonate. That reproduction requires the highest amounts for rescue is particularly interesting given that the HMGR-1 protein is expressed at especially high levels in the spermatheca and that *hmgr-1* mutants produce dead embryos that express high levels of a UPR^er^ reporter, namely *Phsp-4::GFP*. This strongly suggests that the production of healthy embryos requires high amounts of mevalonate. The mevalonate pathway, and in particular its output GGPP, is specifically important for germline development in several organisms, including *Drosophila* and zebrafish [21], [22]. There may be a similar requirement during *C. elegans* germline development.

In summary, we show that the *hmgr-1(tm4368)* mutant recapitulates in a more severe form the effects of statins in *C. elegans* and that different amounts of mevalonate are required for different physiological processes, with reproduction requiring the highest levels.

## Materials and Methods

### *C. elegans* Strains and Cultivation

All genotypes were maintained as described previously [23] and grown at 20°C unless otherwise stated. The Bristol strain N2 was used as wild type (WT). The following alleles and transgenic lines were obtained from the *Caenorhabditis* Genetics Center: *ccIs4251[pSAK2(Pmyo3::NLS::GFP::LacZ); pSAK4(Pmyo3::mtGFP); dpy-20(+)], zcIs4[phsp4::GFP]*, *zcIs9*[*hsp-60::GFP*], *zcIs13*[*hsp-6::GFP*], *atfs-1(gk3094)*, and *hmgr-1(tm4368)*. The *hmgr-1(tm4368)* allele was outcrossed ten times to wild-type worms prior to the experiments described here.

### Preparation of Plates with Additives

Fluvastatin-containing plates were made as previously described [7], [8]. Briefly, 40 mg fluvastatin was dissolved in 2.31 ml dH_2_O. Insoluble components were spun down at 5 000×*g* for 10 min (at 20°C). The supernatant was filter-sterilized and the OD_305 nm_ was measured to determine the final concentration using a standard curve plotted from a known concentration of fluvastatin. Fluvastatin was added directly into NGM media (55°C), to final concentrations of 0.5 mM or 1 mM. Mevalonolactone (Sigma) and paraquat (Sigma) were dissolved in dH_2_O to produce a 1 M and 100 mM stock solutions, respectively; these were added directly into NGM media (55°C) to achieve the desired concentrations.

### Growth Assay

Synchronized L1 larvae were placed onto NGM plates and plates containing different concentrations of mevalonic acid. After 96 hrs, worms were mounted on glass slides, images were acquired in bright field and worm lengths were measured with the ImageJ software (National Institutes of Health) [24].

### Brood Size Assay

Synchronous L1s were plated onto NGM plates containing 20 mM mevalonic acid seeded with OP50. When grown to the L4 stage at least 15 worms were singled out onto new NGM plates and plates containing different concentrations of mevalonic acid. The worms were transferred daily during the fertile period and live progeny were counted 3 days after removal of the hermaphrodite.

### Life Span Assay

Synchronous L4s that were grown on 20 mM mevalonic acid were plated in groups of 5 worms onto NGM and different concentrations of mevalonic acid plates. The worms were transferred every second day during the fertile period and once a week thereafter. All worms were monitored every day and scored as dead when failing to respond upon several touches on the head with the worm-pick.

### Prenylation Assay

The prenylation assay was performed as previously described [7]. Briefly, the plasmid *pGLO-1P::GFP-CAAX* carries the intestinal-specific promoter *glo-1* to express a modified GFP fused to the last 12 aa of the *C. elegans ras-2* gene, including the terminal prenylation motif sequence. The *hmgr-1(tm4368)* mutant that were crossed with the prenylation reporter were placed on 20 mM mevalonic acid and their progeny (L1 larvae) were scored for the number of GFP-enriched intestinal cells 24 hours later.

### Plasmid Constructions

The *hmgr-1P::GFP* transcriptional reporter was constructed by first amplifying 3.06 kb of sequence upstream of the start codon using the primers 5′- GTTCTAGAGCTGAAGATGGGCTAGTTTG-3′ (Xba*I* site underlined) and 5′- GTGGATCCCGCTTATCCGCCACCATAA-3′ (BamHI site underlined) and lysed N2 worms as source of template. The resulting PCR product was cloned into the *pCR-Blunt II-TOPO* vector (InVitrogen), and then subcloned as a Xba*I*-BamHI fragment into the corresponding sites of *pPD95.75* to produce a GFP reporter driven by the *hmgr-1* regulatory region. The *hmgr-1P:: hmgr-1::GFP* translational reporter was similarly constructed but using instead the following primer pairs: 5′- GTTCTAGAGCTGAAGATGGGCTAGTTTG-3′ (Xba*I* site underlined) and 5′- GTGGATCCCATTGTACAACATCTTGTGGC-3′ (BamHI site underlined).

### PCR Detection of the *hmgr-1* Mutation

The *hmgr-1(tm4368)* allele carries a 620 bp deletion that spans the three first exons. The following primers were used to distinguish the wild type and mutant loci: 5′- GGTGCGATCAACATTAGCAA −3′ and 5′- CCACGATTTGTGGATGCAAT −3′ which give a 924 band in wild type and a 305 bp band in the mutant.

### Generation of Transgenic Worms

Germ-line transformation was performed as described by Mello et al. [25] and the dominant *rol-6(su1006)* was used as a marker for transgenic worms. Plasmids were prepared with a Qiagen miniprep kit and used at the following concentrations: *pRF4(rol-6)* of 25 ng/µL, test plasmids of 1 ng/µL and *pBSKS* (Stratagene) of 74 ng/µL.

### Fluorescent and Differential Interference Contrast (DIC) Microscopy

Worms were placed on 2% agarose pads, on glass slides in a drop of 10 mM levamisole as anesthetic and overlaid with a cover slip, then observed with a Zeiss Axio Scope. A1 microscope using a GFP filter or DIC optics. Images were taken using the Axiovision 4.7 program (Zeiss), further processed using Photoshop (Adobe) and quantified with the ImageJ software (National Institutes of Health) [24].

## Supporting Information

Figure S1 **Low amounts of mevalonate have little effect on UPR^mt^.** N2 (WT) and *atfs-1(et15)* worms carrying the *Phsp-60::GFP* transgene were spotted as L1s on experimental plates then scored after 96 hrs.
(TIF)
