# Supplementary figures and images for: Loss of HMG-CoA Reductase in C. elegans Causes Defects in Protein Prenylation and Muscle Mitochondria
Source: PLoS One. 2014 Jun 11;9(6):e100033. doi: 10.1371/journal.pone.0100033 (PMC4053411; doi:10.1371/journal.pone.0100033)

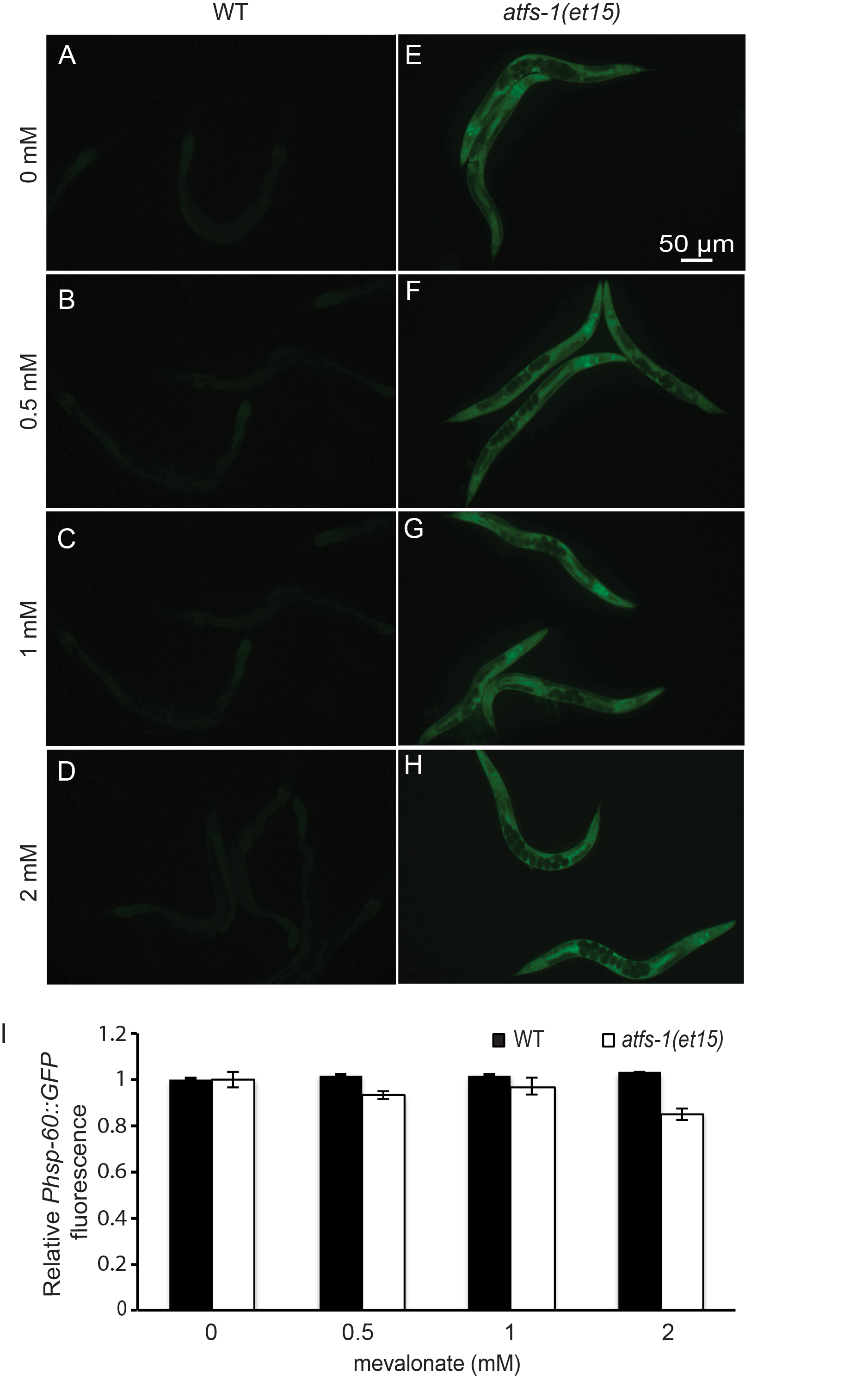

Supplement: Figure S1 — Low amounts of mevalonate have little effect on UPRmt. N2 (WT) and atfs-1(et15) worms carrying the Phsp-60::GFP transgene were spotted as L1s on experimental plates then scored after 96 hrs. (TIF) [file pone.0100033.s001.tif]
